# Recovery of Green Polyols from Rigid Polyurethane Waste by Catalytic Depolymerization

**DOI:** 10.3390/polym14142936

**Published:** 2022-07-20

**Authors:** Rafael Miguel-Fernández, Izotz Amundarain, Asier Asueta, Sara García-Fernández, Sixto Arnaiz, Nora Lardiés Miazza, Ernesto Montón, Bárbara Rodríguez-García, Elena Bianca-Benchea

**Affiliations:** 1 GAIKER Technology Centre, Basque Research and Technology Alliance (BRTA), Parque Tecnológico de Bizkaia, Edificio 202, 48170 Zamudio, Spain; amundarain@gaiker.es (I.A.); asueta@gaiker.es (A.A.); garciafdezsara@gmail.com (S.G.-F.); arnaiz@gaiker.es (S.A.); 2 AIMPLAS Plastics Technology Centre, Parque Tecnológico de Valencia, Gustave Eiffel, 4, 46980 Paterna, Spain; nlardies@aimplas.es; 3 Arcesso Dynamics S.L., Carrer Boters, 3-5, 08290 Cerdanyola del Vallés, Spain; comercial@arcessodynamics.com (E.M.); laboratorio@arcessodynamics.com (B.R.-G.); elenabiancabenchea@gmail.com (E.B.-B.)

**Keywords:** glycolysis, polyurethane, foams, chemical recycling, circular economy, polyols

## Abstract

Polyurethane (PU) is one of the most versatile polymers available and can be found in an infinite number of formats ranging from rigid or flexible foams to elastomers. Currently, most Rigid PU Foam (RPUF) waste is landfilled, even though a small amount is mechanically recycled, in which the material is conditioned in size to a very fine powder, which is introduced as a filler. In this work, chemical recycling of two types of rigid PU foams is studied, the major difference being the aliphatic or aromatic nature of the isocyanate used in the synthesis. A solvolysis process is developed, a chemical depolymerization that breaks the chains by means of a chemical agent, a solvent, in the presence of a catalyst and under controlled process conditions. The glycolysis products are purified by vacuum distillation, centrifugation, and acid water treatment, depending on the most suitable process for each waste type. Optimal process conditions are established to obtain high-purity green polyols by performing a set of catalytic glycolysis reactions at laboratory scale with the previously conditioned RPUF waste samples. The physicochemical properties of the polyols, such as hydroxyl value, acid value, average molecular weight (M_n_), and viscosity, are analyzed. The chemical structure and thermal stability of the polyols are studied by means of FTIR and TGA, respectively. Partial substitution of the commercial polyol (up to 15 wt.%) by the recycled polyols for RPUF synthesis is studied and characterized.

## 1. Introduction

Thermoset Polyurethane (PU) [1] products such as flexible and rigid foams are very versatile materials, long-lasting, and durable and have a wide variety of applications due to their cellular structure and wide range of mechanical properties. TPUs are used as construction materials mostly as thermal insulation, car seats, headliners, acoustic products, instrument panels, headrests, furniture, footwear, coatings, adhesives, sealants, elastomers, and binders [2,3,4].

Polyurethane chemistry is fundamentally based on the condensation reaction between polyols and diisocyanates. These isocyanate groups are very reactive to species with active hydrogens, such as hydroxyl groups, urethane groups, and water. For this reason, during the foaming process of RPUFs, many exothermic reactions of isocyanate groups occur consecutively [5].

In recent years, their recycling has become more urgent due to various reasons such as the high price of raw materials, environmental concern, the closure of landfills, increasing waste disposal rates, increasingly stringent legislative regulations, as well as increased social awareness.

Recycling thermoset PU is a much more challenging process compared with thermoplastic materials. These materials are highly recalcitrant to degradation. Different strategies have been developed for the treatment of production scraps’ end EOL products, such us mechanical recycling, chemical recycling via pyrolysis, solvolysis, or gasification, as well as biological degradation with bacteria, enzymes, or fungi (Table 1).

Landfilling is currently the most applied option to dispose of polyurethane waste, but it is not environmentally acceptable, pollutes the soil, and implies a loss of resources and raw materials [6].

Recycling of polyurethane foams is primarily performed on pre-consumer waste by a mechanical recycling process [7]. Foams are subjected to a process to reduce the size into small flakes or pellets [8,9] and mixed with a binder and processed into parts by compression mainly. The applications for the recycled products are mainly carpet underlays, shock-absorbing mats, and acoustic products. The possible markets are limited, and most of the cases imply downcycling.

Chemical recycling processes include a wide range of options depending on the reagents used and process conditions. In this case, polymer chains are converted into small molecules, which will be different depending on the process. Among the processes studied are glycolysis [10], aminolysis [11,12,13], alcoholysis [14,15] hydrolysis [16,17], gasification, and pyrolysis.

Comparing chemical recycling with mechanical recycling, this is a process with lower costs in terms of both reagents and equipment, but the building blocks obtained after chemical recycling can be used in much more valuable applications in the petrochemical industry or as raw material in the manufacture of new PU resins.

Glycolysis [18,19,20,21,22,23,24] is the most studied process, mainly for pre-consumer flexible foams. Depending on the chemistry used and the nature of the PU, the resulting products are different, being obtained in one or two phases and generating flexible or rigid polyols. These polyols are like those used in the manufacture of PU, which is a great advantage. A limitation of the process is that it is not universal for all types of PU, but must be segregated by typology. The glycolysis of RPUFs implies the treatment of the residues with a low-molecular-mass glycol and temperature. A homogeneous single-phase product with low viscosity and high hydroxyl value is obtained and can be used as a partial substitute for commercial polyether polyols in the synthesis of new rigid foams [25,26,27].

The hydrolysis process involves the reaction of PU with water, in liquid or gaseous form. Polyols, amine-type intermediates, and carbon dioxide are produced. The polyols can be used in the manufacture of new PU, and the amine intermediates after a purification process can be used as isocyanate. The hydrolysis process has not been developed on a large scale due to the energy and/or pressure requirements, which make it economically unfeasible.

Gasification is a highly exothermic process that produces a mixture of carbon monoxide and hydrogen, called synthesis gas, as well as ash. An advantage of gasification is that no segregation or separation of waste is required prior to the process. However, the economic viability is limited by the need to manage the ash generated and to purify the gases, as well as being sensitive to the availability of uses for syngas as a feedstock for the generation of products from the gas mixture generated.

In the case of pyrolysis, polymer chains are thermally decomposed into small hydrocarbons, generating liquids, gases, and char. An advantage of this process is the small amount of waste generated in the form of ash and the possibility of using the products generated in the petrochemical industry. However, the requirements of this industry to accept pyrolytic oils are very restrictive with respect to the compositions accepted, with high needs for purification of gases and upgrading of liquids.

Energy recovery is one of the least desirable options as it prevents the recovery of raw materials, although in some highly contaminated waste scenarios, it may be an option to avoid landfilling, if the purification of the emitted gases is carried out properly.

Biological degradation can be carried out by micro-organisms, enzymes, or fungi. Although these are promising processes, they are at a very preliminary level of development, at the laboratory scale, and cover very specific stages of the process. These processes are usually very slow and have a low yield, although they do not require chemical and thermal conditions to be used.

The recycling of rigid polyurethane foam waste has been less studied than flexible ones, and the number of references on post-consumer foam is even lower. Morooka et al. [18] studied the glycolysis of RPUFs from refrigerators using Diethylene Glycol (DEG) as the solvent and BaO or Diethanolamine (DEA) as the catalysts. They added the outcoming glycolysate to a commercial polyol (up to 10 wt.%), and new RPUFs were produced; their thermal conductivities and compressive strength measurements were similar to virgin foams. Nikje and Nikrah [28] studied microwave irradiation at atmospheric pressure to assist glycolysis reactions of RPUFs by microwave irradiation to the waste instead of heating the vessel. Low-weight glycol DEG was used, and, as catalysts, various ones were used such as sodium hydroxide (NaOH), potassium hydroxide (KOH), sodium Acetate (NaAc), and Zinc Acetate (ZnAc2). The reactions were carried out with a DEG to PU foam ratio of 2:1. Results showed that the optimal catalysts were sodium hydroxide (NaOH) and potassium hydroxide (KOH), whereas the worst one was Zinc Acetate (ZnAc2), due to that the glycolysis reaction time being very high when using this catalyst. New rigid foams were fabricated using the single-phase products with up to 40 wt% substitution of virgin polyol. Zhu et al. [20] studied the glycolysis of rigid foams from refrigerators, and the results showed higher yields by using Ethylene Glycol (EG) instead of DEG. The optimal reaction conditions were an EG:PU ratio of 1:1 by mass, a catalyst concentration of 1 wt.%, a temperature of 198 °C, and a reaction time of 2 h. In the new foam formulations, the recovered polyols were incorporated up to 10 wt.% with respect to the total amount of polyol.

Although previous research has identified glycolysis as one of the most suitable chemical recycling processes for PU recycling, applications developed from those findings mainly focus on clean flexible PU foams with a known composition. The recovery of polyols is easier, and higher purity can be achieved due to a low-intensity post-treatment required by glycolysis products of flexible PU. In the case of rigid PU, the reaction product is a single phase, and for that reason, the recovered polyols are mixed with the solvent and with other chemical by-products of the reaction and compounds derived from the composition of the PU waste. Hence, it is necessary to concentrate the recovered polyols and validate their use in the synthesis of new PUs. Furthermore, the present work deals with real PU waste that is currently being generated and landfilled in large quantities, so this paper aims to present a technically feasible solution based on the principles of the circular economy, thus allowing the manufacture of new value-added polyurethanes and closing the cycle of PU material through the application of a chemical recycling process [29].

The purpose of the research is to understand the steps involved in the chemical recycling of post-industrial complex waste RPUFs for housing and bracket applications and the synthesis of new recycled foams based on recycled polyols. After purifying the reaction products by vacuum distillation, the recycled polyols were incorporated into new formulations of RPUFs. The recovered polyols were analyzed through various characterization techniques to examine their composition. Certain compounds, such as amines, that significantly influence the foaming process were also identified and quantified. Foam synthesis reactions were monitored, performing an analysis of the reactivity and exothermicity of the process. Temperature profiles and characteristic times of the reactions were observed as a function of the amount of recycled polyol incorporated. Physical, chemical, and mechanical properties of interest were compared to conventional foams.

## 2. Materials and Methods

### 2.1. Materials

**Figure 1:**
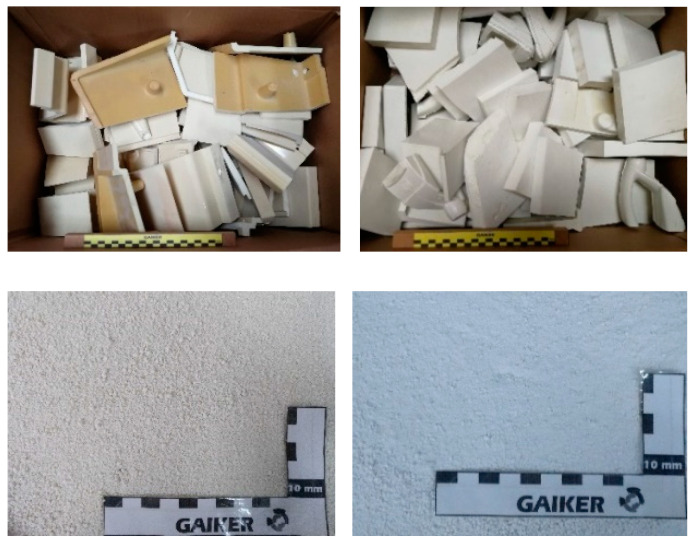
RPUF B: medium-/high-density (650–750 g/m^3^) ochre-colored expanded rigid PU pieces (above level) and milled (below level), RPUF C: high-density expanded rigid PU parts, bi-density solid surface type (850 g/m^3^), white color with micronized alumina and titanium oxide fillers (above right) and milled (below right).

Two different industrially produced RPUF pieces for housing and bracket applications, manufactured and provided by the company Arcesso Dynamics S.L. (Barcelona, Spain), were considered for this work and named RPUF-B and RPUF-C (Figure 1). RPUF-B is composed of a 50 wt.% aromatic polyisocyanate MDI-based (4,4 diphenyl methane diisocyanate) and 50 wt.% of three base polyols, foam stabilizers, amine catalysts, and other additives. Polyol 1 is a glycerin-based tri-functional polyether polyol; Polyol 2 is a sucrose-based high functional polyether polyol; Polyol 3 is ethylenediamine and propylene oxide-based tetra-functional polyether polyol. A foam stabilizer (polysiloxane-polyether), amines (diethanolamine and triethanolamine), additives (anomeric polyphosphate as flame retardant) and surfactants and expanders were added.

RPUF-C is composed of an aliphatic polyisocyanate type HDI: 1.6 hexane polyisocyanate and of tri-functional polyether polyol with high reactivity and molecular weight. Dibutyl Dilaurate (BDTL) was used a as a catalyst and mineral fillers (micronized alumina and titanium oxide), surfactant additives, and anomeric polyphosphate as flame retardant and expanders (water and/or liquid gas and/or gas encapsulated in polymeric microspheres).

Ethylene Glycol (EG) or Diethylene Glycol (DEG) were used as solvents and NaOH, Na acetate, or Diethanolamine (DEA) as catalyst for glycolysis.

### 2.2. Glycolysis Reactions of Rigid PU Foam Wastes

Rigid polyurethane foam wastes were milled by means of a blade mill to different particle sizes (5 mm, 2 mm, and 0.5 mm in diameter) prior to the glycolysis reaction. The selected glycol was fed into a 500 mL three-necked glass reactor, equipped with a stirrer that was set at 100 rpm, a thermometer, and a reflux condenser. Ethylene glycol or diethylene glycol as the glycolysis reagents and NaOH, Na acetate, and Diethanolamine (DEA) as the catalysts were added at different mass ratios given in Table 2 (for RPUF-B) and Table S1 (for RPUF-C) and preheated to the boiling temperature of the glycolysis reagent used. Finally, waste PU pieces were fed. During the reactions, intermediate samples were taken at different times for further analysis. After the reaction, the unreacted solid phase of the solution was separated from the liquid phase by two types of processes, depending on the characteristics and amount of solid remaining: pressure filtration (0.7 µm filter) and/or centrifugation. The filters were dried at 40 °C, and the conversion was calculated for each reaction.

### 2.3. Purification of Glycolysis Products

The product of the glycolysis reaction has excess solvent, so a purification step is necessary. The final reaction product is distilled by means of vacuum distillation in a rotary evaporator, under a vacuum close to 50 hPa, and an oil bath at 140 °C, for approximately 3 h and 30 min, or purified with extraction of acid water solution (80 °C for 2 h) to remove the amines formed during the glycolysis. Finally, two products were obtained: the distilled glycolysis agent and the recycled polyol, which were collected separately to analyze them and, in the case of the polyol, to use it in the synthesis of new RPUFs.

### 2.4. Characterization Techniques

#### 2.4.1. Waste Characterization and Monitorization of Reaction Evolution

GPC: Measurements were carried out on a Shimadzu chromatograph, equipped with two piston pumps, an automatic injection system, a rack for 150 vials, an electric oven, two Waters HR-2 (pore size 500 Å) and HR-0.5 (pore size 50 Å) columns, and a refractive index detector. The column set allows the detection of molecular weights between 0 and 20,000 g/mol, thus suitable for the analysis of polyols and derivatives of lower molecular weight. The whole equipment is controlled by a computer system with the CLASS VP 4.2 software, which controls, records, and analyses the results.

The result is expressed as the molecular weight distribution, molecular weight, Mn, Mw, and Mz, as well as the polydispersity index.

TGA: Thermogravimetric Analysis (TGA) is a technique used to determine the thermal stability of a material and its fraction of volatile components by observing the change in mass that occurs when a sample is heated at a constant rate. Thermogravimetric tests were carried out using the Mettler Toledo TGA/DSC 1 Stare System and data processing with TA STARe Evaluation Software. They were carried out at 20 °C min^−1^ from room temperature up to 600 °C and in a controlled atmosphere of N_2_.

DSC: The Differential Scanning Calorimetry (DSC) technique was used to study the thermal transitions of the polymers, using the DSC-1 equipment (Mettler Toledo). Prior to the analysis, the sample was heated and cooled to eliminate the thermal history of the polymer. Subsequently, the samples were heated from room temperature to 300 °C, with a ramp of 10 °C min^−1^ and a nitrogen flow of 15 mL min^−1^.

ATR-IR: The chemical structure of the products recovered from the glycolysis reaction was studied by Fourier-Transform Infrared Spectroscopy (FTIR). This analysis allows the identification of the characteristic absorption bands of the main compounds present in the samples. The products obtained in all reactions were analyzed by infrared spectroscopy, using the Shimadzu-Miracle 10 IR affinity 1 instrument with the Single Reflection ATR accessory and the IR Solution data processor. Twenty scans were performed at a resolution of 4 cm^−1^ in the region 4000 to 400 cm^−1^.

Refraction index: The refractive index determines how much the path of light is deflected or refracted as it enters a material. This is described by Snell’s law of refraction [30]. Refractive index measurements were performed with a digital hand-held “pocket” refractometer, PAL-RI from ATAGO, with a refractive index measurement range between 1.3306 and 1.5284. At different reaction times, the refractive index measurements were taken as a tool to assess the progress of the reaction.

XRF: The residues were analyzed by X-Ray Fluorescence (XRF) using the Innov-X Systems Alpha series analyzer with a metal-ceramic tube and tungsten filament. This technique was used to determine trace elements present in the polymers from additives such as plasticizers, foam stabilizers, or pigments and/or the catalysts used for their manufacture.

Solid content: A sample of each waste was calcined in a muffle at 625 °C for 5 h, according to UNE EN ISO 3451-4, to oxidize all organic matter present in the sample and to determine the content of inorganic matter (metals, oxides, etc.).

#### 2.4.2. Characterization of Polyols

ATR-FTIR: The products obtained in all reactions were analyzed by infrared spectroscopy, using the Shimadzu-Miracle 10 IR affinity 1 instrument with the Single Reflection ATR accessory and the IR Solution data processor. Twenty scans were performed at a resolution of 4 cm^−1^ in the region 4000 to 400 cm^−1^.

TGA: Thermogravimetric (TG) tests were carried out using the Mettler Toledo TGA/DSC 1 and data processing with TA STARe Evaluation Software. They were carried out at 20 °C min^−1^ from room temperature up to 600 °C and in a controlled N2 atmosphere.

GPC: The equipment used was a WATERS HPLC 1525 equipped with Autosampler 717 PLUS with detectors: WATERS 2424 ELSD (evaporative light scattering detector). The column: TOSOH Bioscience TSKGEL GMHHR-H 7,8 mm ID and 30 cm length. Dissolution of samples in THF. Concentration of samples: approximately 0.5%. Elution solvent: THF (0.8 mL/min). Universal PS standards were used: 98,900 g/mol, 37,200 g/mol, 2500 g/mol, 1302 g/mol, 418 g/mol. The data were analyzed with Empowe, and each vial was injected twice; the molecular weight distribution was calculated based on PS standards of different molecular weight.

Screening: Gas chromatograph GC 6890N coupled to mass detector 5975, both from Thermo, with capillary column HP-5MS (30 m × 0.25 mm × 0.25 μm). This technique was used to analyze the composition of PUR glycolysis products (amines, % of polyols, etc.).

Hydroxyl number: The hydroxyl number (HOI) of a polyol is defined as the mg of potassium hydroxide equivalent to the hydroxyl groups present in 1 g of sample. The determination of the index is carried out by an adaptation of the test method described in ASTM E1899-16. An automatic titrator from Mettler-Toledo, Model DL25, as used for the analysis.

### 2.5. Synthesis of New RPUFs

The feasibility of incorporating different green polyols obtained from the glycolysis reaction of rigid PU in a new polyurethane synthesis cycle was evaluated. For this purpose, different percentages of the green polyols were incorporated into the formulation. The materials evaluated were:

- Recycled polyols from the glycolysis of polyurethanes with aromatic isocyanates (RPUF-B).
- Recycled polyols from the glycolysis of polyurethanes with aliphatic isocyanates (RPUF-C).
- Recycled pulp obtained as a by-product of RPUF-C recycling, evaluated as a possible filler in new formulations of aliphatic polyurethanes.

First, the reaction onset times (time taken for the reaction to start once all reagents are added) and the curing time once foaming has started were evaluated using a disposable beaker.

For the realization of polyurethane plates at laboratory scale to evaluate the quality of the foam, a clean mold was used, and the mixture of all the reagents was left to cure inside the mold for 20 min. After this time, the mold was cooled and opened, and the part was easily removed.

## 3. Results and Discussion

### 3.1. Characterization of Aromatic (RPUF-B) and Aliphatic (RPUF-C) PU Wastes

Inorganic matter content after muffle calcination of the wastes is shown in Table S2.

The Attenuated Total Reflection Infrared Spectroscopy (ATR-IR) technique allows the identification of the characteristic absorption bands of the main compounds present in the samples. It was applied to the ashes of the two samples from calcination. Previously, the samples were dried for 48 h at 60 °C to remove the moisture.

The analysis of the bands was based on the observation of their intensity, shape, and position in the IR spectrum (Figure S1). In the two samples, the characteristic bands of polyurethane can be identified: around 3329–332 cm^−1^, the characteristic band of the tension vibration of the –N–H bond associated through hydrogen bonds is observed; at 2855 and 2940 cm^−1^ are the two characteristic peaks of the symmetric and asymmetric vibrations of the –CH_2_ bonds, respectively. Around 1750 cm^−1^ appears the band characteristic of the tension vibration of the carbonyl group –C=O. At wavelength 1540 cm^−1^, the characteristic band of the coupling of the symmetric deformation vibration of the –N–H bonded groups with the tension vibration of the –C–N groups is seen. In turn, the band at 1224 cm^−1^ is associated with the coupling of the deformation vibration of –N–H with the tension vibration of –C–N in the urethane group.

The IR spectra of the ashes resulting from the calcination of the residues are shown below (Figure S2), and silica-silicate bands are observed. In the IR spectra of RPUF-B and RPUF-C ashes, the characteristic bands of the silicates can be observed at 1080 cm^−1^ for the asymmetric (Si–O–Si) vibrations, at 950 cm^−1^ for the asymmetric (Si–OH) vibrations, and at 800 cm^−1^ for the symmetric (Si–O–Si) vibrations.

The TG and dTG curves of the residues show a two-stage degradation of RPUF-B and RPUF-C (Table S3), as occurs in most of the literature studies under the same operating conditions. The first mass loss peak occurs between 250 and 350 °C and the second between 350 and 550 °C for the samples, whose curves are similar to each other. The first stage (250–350 °C) can be attributed to the degradation of the rigid segments of the PU, as a consequence of the relatively low thermal stability of the urethane groups, whose degradation depends on the nature of the isocyanate group. The polymer chain breaks down, giving rise to isocyanates, alcohols, amines, olefins, and carbon dioxide. The second stage (350–550 °C) can be associated with the decomposition of the soft segments and fragments obtained in the previous degradation to smaller molecules.

**Figure 2:**
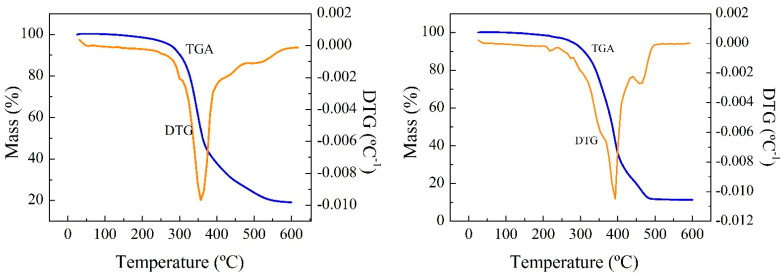
TG (blue) and dTG (orange) curves of the residues RPUF-B and RPUF-C.

Then milligrams of the sample was used, with thermobalance heating from room temperature to 600 °C, ramped at 10 °C min^−1^ under constant N_2_ flow. Mass loss as a function of temperature was continuously monitored and is plotted in Figure 2.

The solid remaining after thermal degradation of the sample in the N_2_ atmosphere is a solid formed by the inorganic fillers and a carbonaceous residue, called char, coming from the pyrolysis of RPUF.

**Figure 3:**
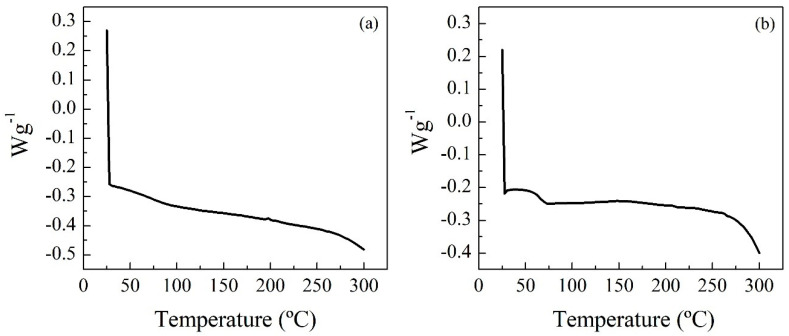
DSC curves of RPUF-B (**a**) and RPUF-C (**b**).

The Differential Scanning Calorimetry (DSC) technique (Figure 3) was used to study the thermal transitions of the polymers. Prior to the analysis, the sample was heated and cooled to eliminate the thermal history of the polymer. Subsequently, the samples were heated from room temperature to 300 °C, with a ramp of 10 °C min^−1^ and a nitrogen flow rate of 15 mL min^−1^. The glass transition temperature is an indication of the stiffness/flexibility of the polymer: the higher the stiffness, the higher the glass transition temperature is. This indicates that the stiffness of the tested samples decreases in this order: RPUF-B > RPUF-C. In the case of the last residue, its glass transition temperature was not found in the temperature range studied (25–300 °C).

The residues were analyzed by X-Ray Fluorescence (XRF) to determine trace elements present (Table S4) in the polymers from additives such as plasticizers, foam stabilizers, or pigments and/or the catalysts used for their manufacture.

### 3.2. Glycolysis of Aromatic (RPUF-B) and Aliphatic (RPUF-C) PU Wastes

**Figure 4:**
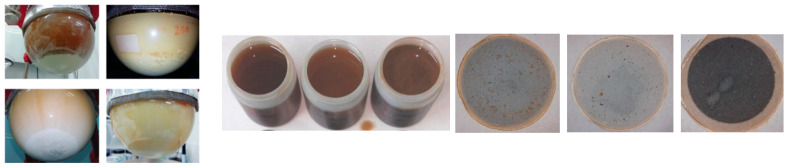
Example of glycolyzed products at 2 h of PU reaction: RPUF-B (**left**); RPUF-C (**right**). Liquid phase R5, R6, R7. Solid phase non-reacted material after filtration R5, R6, R7.

Firstly, reactions without catalyst were studied, and it was observed that RPUF-B shows high conversions (>90%) at 2 h. However, the need to introduce a catalyst in RPUF-C was observed. NaOH catalyzes the reaction, slightly improving the conversion of RPUF-C, as does sodium acetate. However, the latter makes the reaction unstable during the first minutes and, in the case of RPUF-C, significantly reduces the temperature. For this residue, the use of conventional catalysts such as DEA was studied. However, their use significantly reduces the conversion. The use of EG versus DEG allows, in general, obtaining more manageable glycolyzed products, with lower viscosity, which makes possible the subsequent filtering and/or centrifugation stages. In the case of RPUF-C, DEG is a solvent that prevents the glycolysis reaction, unlike EG. As for temperature, the greatest effect was observed for the RPUF-C residue. The reaction at 180 °C did not comply with the mass balance, so it was not possible to calculate the conversion; however, a large amount of solid residue was observed, and qualitatively, a much lower conversion compared to the reaction at 198 °C can be appreciated (Figure 4).

### 3.3. Purification, Analysis, and Characteristics of Polyols from Aromatic (RPUF-B) and Aliphatic (RPUF-C) PU Wastes

The conversion obtained in the reaction was calculated as a function of the weight of RPUF reacted per weight of residue fed. The main parameters studied were the type of catalyst, the solvent (EG: Ethylene Glycol and DEG: Diethylene Glycol), the reaction temperature, and, in some cases, other parameters such as reaction time, the ratios of RPUF—solvent, and catalyst—RPUF.

The amount of polyols obtained was calculated as the sum of the areas of the peaks obtained by GPC, after comparing them with the peaks obtained from the reference polyols.

**Figure 5:**
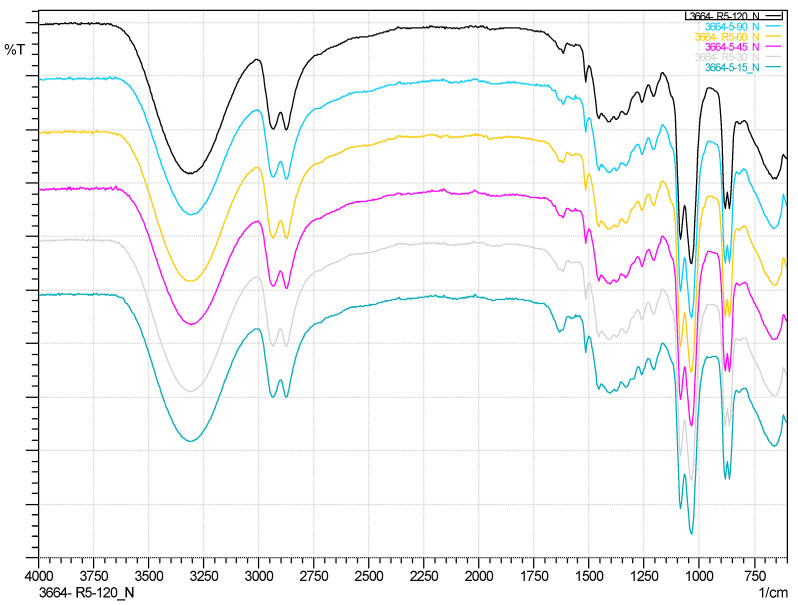
ATR-IR spectrum of RPUF-B glycolyzed products (200 ºC, EG, NaOH, 0.002 g/mol PUR) at different times (15, 30, 45, 60, 90, and 120 min).

The possibility of using IR spectroscopy to monitor the conversion of RPUF was evaluated. An example of the evolution of the spectra over time for one of the residues is shown in Figure 5. However, the EG bands make it difficult to distinguish the peaks of the products, and it is not possible to observe appreciable changes in the different spectra.

The products of the glycolysis reactions obtained were characterized, and a purification protocol was established, by unreacted solid phase separation.

Since the absorption bands of the solvent overlap with those of the compounds formed during glycolysis, preventing the observation of their real spectra, it was decided to remove the solvent EG from all reactions by distillation in a rotary evaporator and to analyze them by infrared spectroscopy.

In Figure S3 ATR-IR spectrums of comercial reference polyols are shown.

In Figure S4 is compared the ATR-IR spectrum of Reaction Number 5 at 120 min compared with EG and PURF-B; it is clearly seen how the carbonyl group disappears. Figure 5 shows how the reaction evolves as time progresses. The need for the use of catalyst is observed in Figure S5, where a yield to liquid of 98% is achieved; on the other hand, in Reaction 19, where no catalyst was used, the carbonyl peak and a lower yield are observed.

ATR-IR spectra of PURF-B and PURF-C of the refined products of the reactions under similar conditions of operation show that the carbonyl group disappears in the case of PURF-B and remains in the case of PURF-C (Figures S6 and S7).

Thermogravimetric (TG) tests were carried out at 20 °C min^−1^ from room temperature up to 600 °C and in a controlled N_2_ atmosphere. The spectra show that for RPUF-B, there is a decrease in the decomposition temperature with time, which is consistent with the breakdown of the RPUF polymeric chain, while the opposite happens for RPUF-C.

**Figure 6:**
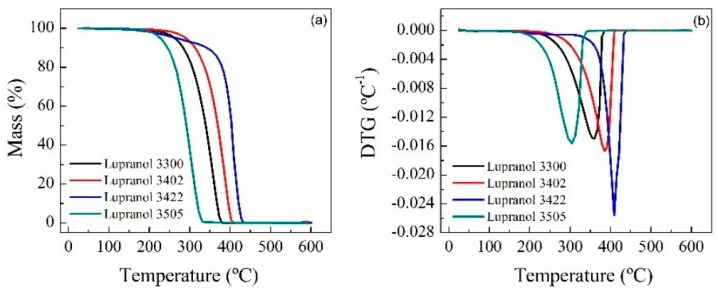
TG (**a**) and dTG (**b**) of reference polyols.

**Figure 7:**
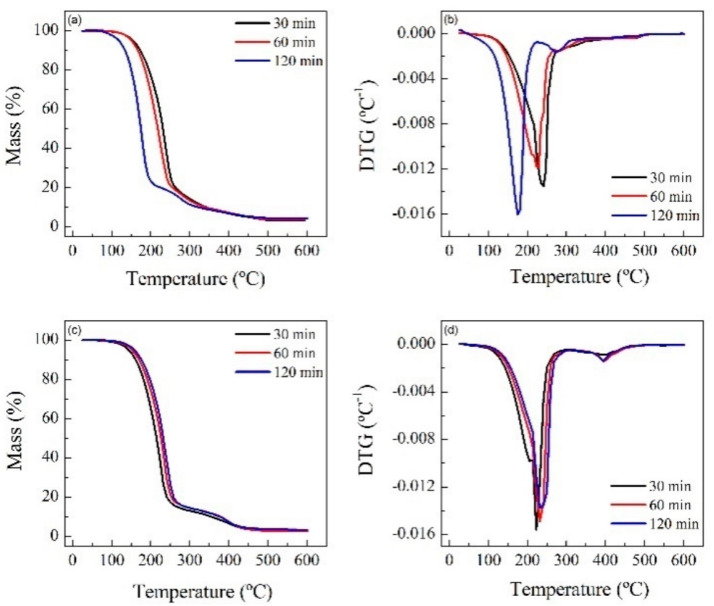
TG and dTG analysis of the products obtained in the reactions at 200 °C, 2 h, PUR/solvent ratio = ¼, and NaOH catalyst (0.002 mol/g) of the residues: RPUF-B (**a**,**b**) and RPUF-C (**c**,**d**).

TGAs of the reference polyols and some reactions were carried out in order to establish some relationship between the properties of the materials (Figure 6 and Figure 7).

**Figure 8:**
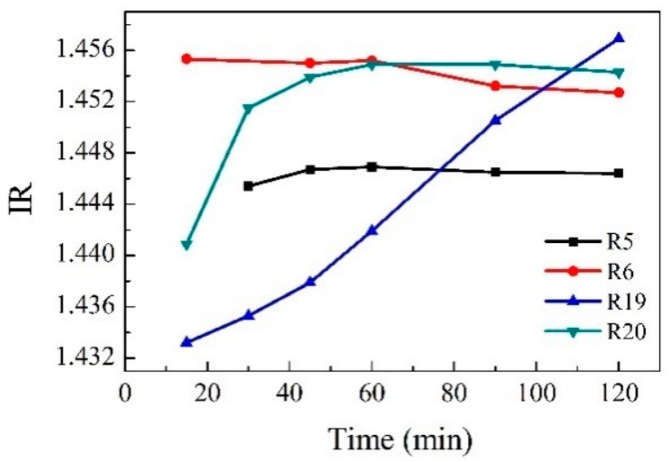
IR of Reactions 5, 6, 19, and 20 at different times.

Refractive index measurements as a method of reaction monitoring were studied, and Figure 8 shows how the refractive index varies as the reaction progresses depending on the conditions such as different temperatures or the type of catalyst. Reaction R19 (blue) was carried out without catalyst. Its corresponding curve shows that the change in refractive index versus time is progressive, indicating that the polymer is continuously dissolving. On the other hand, in reactions R5, R6, and R19, the modification of the refractive index is very fast in the first minutes of the reaction and then remains constant; this could indicate a fast depolymerization. The measurement of the refractive index as a method for monitoring a catalytic depolymerization reaction has not been reported in the literature.

The chromatograms corresponding to the first series of GPC analyses are shown below, with standard samples (commercial polyols) and samples from different reactions previously detailed (containing a mixture of polyols, ethylene glycol, and by-products). The area of the identified compounds is given by the trapezium method and by the standard integration method of the GPC equipment. These values are detailed in Figures S8–S11.

As indicated above, the results of the GPC analyses performed indicate that complete depolymerization of polyurethane residues into diols (and other polyurethane decomposition products generated in parasitic or parallel reactions) occurs in very short reaction times (Table 3). This indicates the fast kinetics of the glycolysis of polyurethane residues under the above-mentioned operating conditions. Furthermore, a significant concentration of the ethylene glycol used as the solvent in the solvolysis reaction is observed.

In order to further study the kinetics of the reaction and to evaluate the evolution of the depolymerization reaction at short times, reactions were carried out at 1, 3, 5, 7 and 10 min. GPCs were performed at these times in order to define the kinetics of the reaction (Figure S12).

**Figure 9:**
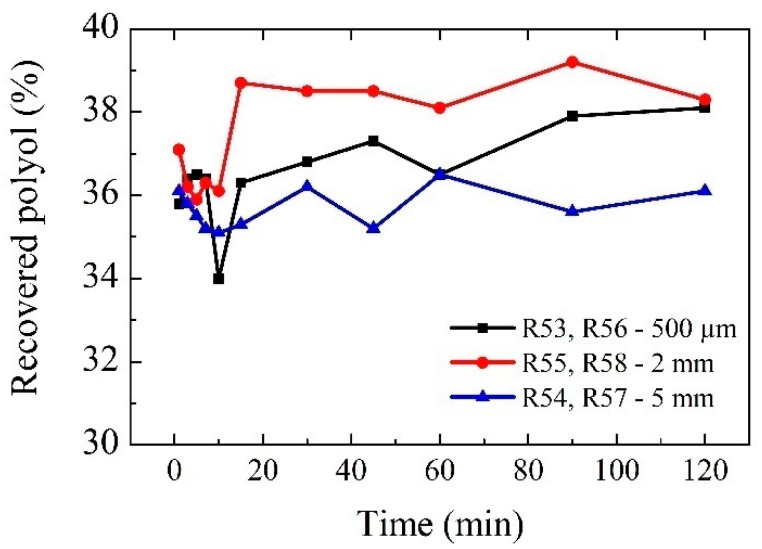
Concentration in percentage of recovered polyol at different times.

The analysis of the GPCs performed at the different times indicates that in the case of the RPUF-B residue, the reaction is practically instantaneous. This indicates that as the PU residue in question is being fed to the solvent in the reactor at boiling conditions and with the appropriate catalyst, depolymerization of the PU is occurring immediately. Similarly, the results of the GPCs show that the generation of polyols is very similar at all times when aliquots were taken for analysis. In other words, not only is depolymerization immediate, but the generation of polyols in relevant concentrations also occurs in short reaction times, as can be concluded from the GPC analyses performed. Furthermore, the GPCs performed indicate that polyols of a similar structure to the original ones used in the formulations of the initial polyurethanes (Lupranols) are obtained, since equivalent peaks are obtained in similar retention times comparing the GPCs of the reaction products with those of the reference polyols (commercial polyols). Noteworthy also is the presence of other peaks in the GPCs, which indicate the presence of reaction products that are additional compounds to the starting polyols. The chemical structure of these compounds is associated with the isocyanate commonly used, as well as fillers and additives, in the manufacture of polyurethane. The results obtained are shown below in Table 4 and Figure 9.

The influence of particle size on the progress of the reaction was studied. For this purpose, three different PU depolymerization reactions were carried out, named: 53 and 56, 54 and 57, and 55 and 58 for RPUF-B and R47 for RPUF-C, operating with a particle size of 500 microns, 5 mm, and 2 mm, respectively. Aliquots were taken at different reaction times and analyzed by GPC.

The study of the samples RPUF-C by screening to see if the reaction time and particle size of the residue influenced the amine content was useful, and the results are shown below (Figures S13–S15). It is interesting to note that amines are only formed when the glycolysis reaction is taken up to 120 min, and in the case of the 5 mm residue, not even at 120 min are amines formed.

The amines of the GPs obtained from RPUF-C waste glycolyzed at 120 min were highly reduced by purification (through extraction in heated acidic water). The reaction time trials of new PU foams’ formation with these GPs after amine purification showed that GPs continue to catalyze the polymerization reaction. GPs were characterized by elemental analysis, and it showed a high amount of aluminum (8895.17 mg/kg RPUF-C versus 37.72 mg/kg for RPUF-B). So far, it can be concluded that it is not the amines that catalyze the polymerization reaction of PUR, but the high aluminum content of the initial RPUF-C waste.

Regarding the evolution of the depolymerization reaction of the glycolysis reaction of RPUF-C, it could not be monitored by IR or HPLC as the initial PUR is not in the reaction medium, but in the solid state. Therefore, the total amount of polyols obtained from glycolysis and after centrifugation and distillation was studied to quantify the amount of polyol formed and to give information to know the optimum reaction time and granulometry. The results are shown in Table S5, always starting from 100 g of RPUF-C residue. In view of these results, 5 mm was selected as the optimum granulometry, and the glycolysis reaction was conducted at lower times (5 and 30 min); the results showed a quantity of polyols lower than 60 min, then it can be concluded that the optimal glycolysis reaction for the RPUF-C residue is 5 mm grind waste for 90 min glycolysis. Although 500 microns of the residue at a 120 min glycolysis reaction formed a bigger quantity of polyols, this was not considered as it is not worth increasing the time and energy required to grind the residue to 500 microns and to leave the glycolysis 30 min longer.

After purification of the glycolysis products by vacuum distillation, the polyols obtained by the techniques explained in the previous section were analyzed. In addition, Commercial Polyol (CP) of petrochemical origin was also analyzed. The values of the hydroxyl number (HOI), acid number (AI), and viscosity (μ) of the polyols are shown in the following Table 5.

On the one hand, the recovered green polyols showed significantly higher hydroxyl index values than the commercial polyol as a consequence of the presence of glycolysis by-products and ethylene glycol (IOH of the EG: 1807.6 mg KOH/g) remaining in the polyol after the purification of the glycolate. On the other hand, the AI was higher in the recovered polyols compared to the acidity of the commercial polyol, although it did not exceed the maximum acidity value (10 mg KOH/g) that polyols should possess. Since the acid number mainly indicates the concentration of carboxyl groups present in the polyols obtained, some limitation of the glycolysis of the residual PUR foams was observed. It should be noted that the recovered polyols are much more viscous than the original commercial polyol. The large difference in these values is possibly due to the higher number of hydrogen bonds present in the green polyols, directly related to a higher hydroxyl index (higher amount of OH groups) of the recycled polyols. Although the viscosity of the recovered polyols is high, it was similar to the values of commercial polyols used in the synthesis of PUR foams.

The hydroxyl number of each polyol obtained was measured (see Table S6). As is shown, the HOI did not follow a tendency.

### 3.4. Results of RPUFs Synthesized from GP

#### 3.4.1. Preliminary Trials

Preliminary trials of the introduction of GP-B (from RPUF-B) and GP-C (from RPUF-C) into aromatic (RPUF-B) and aliphatic (RPUF-C) formulations were tried. Initially, the amount of GP in the total composition of polyols was 5, 10, and 15%. The results showed a reduction of the standard reaction initiation time. For concentrations higher than 5% of GP, the reaction mixtures of the PUR formulation were unmanageable in terms of reaction times and texture, as the system gelled and formed an inhomogeneous, high-density paste that was very difficult to inject into the laboratory mold. In the case of GP-C, the concentration was reduced to 2.5% to obtain a mixture able to make PUR plates. Plates of RPUF-B with 5% of GP-B and 2.5% of GP-C, as well as plates of RPUF-C with 5% of GP-B and 2.5% of GP-C were prepared.

#### 3.4.2. Using Purified GPs

In view of the results obtained and especially the reduction in PUR reaction initiation times, the GPs were purified to reduce the amine content. The properties of these GPs as shown in Table 5. A new battery of samples was carried out with these new GPs following the same experimental structure as in the previous cases. The introduced percentage of the GPs was 5%. It was observed that the mixture for aromatic RPUF-B reacted very fast; before reaching the mold, the material had started to react, and only half of the reaction mixture could be introduced. It was also observed that the foaming was very high. It was decided to repeat the test by increasing the amount of GPs and eliminating the amine catalyst and water as a foaming agent, but even so, the standard formulated polyol blend with aromatic isocyanate reacted very fast. In all cases, the reaction started before the end of stirring, and therefore, it was impossible to inject before the start of the reaction. The resulting plates, although looking good, were of very low density and highly brittle.

**Figure 10:**
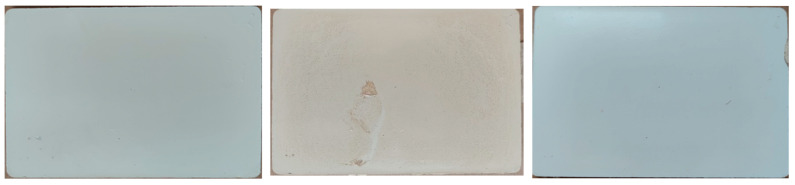
Plates of 5% (**left**) and 15% (**center**) of GP-B in RPUF-C formulations. Plate of 5% of GP-C in RPUF-C formulations (**right**).

The same experiment was developed for the fabrication of plates with the aliphatic RPUF-C formulation. With 5% of GP-B, the reaction was not as fast as the first batch of GPs (without purification), then several plates (see Figure 10 left) were made at this concentration, increasing it until 15% (mixtures of 85% standard polyol and 15% GP-B); in this case, the reaction was much faster and a clear separation appeared in the reaction mixture; this was reflected in the surface defects present on the plates obtained; it was therefore decided not to increase the concentration of GP higher than 15%. Plates were prepared also using 5% of GP-C (Figure 10 right). In this case, unlike the previous tests, it was possible to inject the polyol-isocyanate mixture because it did not increase in viscosity as quickly. The parts had standard densities and showed no surface defects, except for the slightly beige color. As the concentration of GP-C increased, the PUR reaction mixture gelled rapidly, preventing it from being injected into the mold.

As a conclusion based on the previous results obtained with the aromatic RPUF-B formulations (very fast reactions, non-homogeneous materials, and difficult-to-handle reaction mixtures), it was decided to work with the subsequent batches by introducing the GP in a low concentration only in the more manageable aliphatic RPUF-C formulations.

#### 3.4.3. Final Study of Free Foaming and Plate Defects of GPs and Paste in RPUF-C Formulations

In this last stage, two main types of experiments were carried out to evaluate the properties and reactivity of the GPs together with the standard polyol: firstly, a free foaming test, to measure more accurately the reaction initiation time, to appreciate the homogeneity of the mixture, the curing time, and the visual appearance of the resulting material and, secondly, a lab-scale PUR plate that allowed characterizing the final material in terms of density, visual appearance, and molding.

GP-B is dark brown in color, creamy in texture, and has good solubility; however, GP-C is a solid (see Figure S16). Both were incorporated into Component A of the RPUF-C formulations in two different concentrations (5 and 10%) for GP-B and 5% for GP-C. All three GP-C components were subjected to several cycles of temperature and agitation because they presented difficulties being dissolved together with the standard formulated polyol. This dissolution did not occur in the case of the GP-C processed at 500 microns.

In Table 6, it can be observed that the behavior of GP-B was different from GP-C, as the higher the concentration of GP-B, the slower the initiation of the reaction was, with the same amount of catalyst, which seemed to indicate an inhibiting effect on the polymerization reaction of RPUF-C. In the case of GP-C, the behavior was within the reaction parameters of the aliphatic standard RPUF-C, with reaction times around 50–60 s and curing times close to one and a half minute. The foamed material obtained was homogeneous and slightly brown in color (GP-B) and white in color (GP-C).

Plates were made with the incorporation of the 5 and 10% GP-B and 5% for GP-C. In all cases, the reaction mixture was stable enough for manual injection into the mold, and plates with good density were obtained. Regarding the surface, all had a good appearance, except for GP-B at 10%, which showed a brittle appearance and surface defects, such as rash or weakness against scratching (see Figure S17 left). For GP-C, localized brown spots were observed on the surface of the plates formulated with GP-C at 5 mm (Figure S17 right), which could indicate that the recovered polyols were not fully incorporated into the standard polyol.

Since the initial aliphatic PUF-C waste contains a high percentage of mineral fillers, after processing by glycolysis, a by-product (the solid that remains after filtering the glycolysis mixture) is a paste of recycled origin, which is mostly composed of these mineral fillers, but also, in a very small percentage, of initial PUF-C that has not degraded after glycolysis. Because of this, experiments were carried out to evaluate whether this paste could be incorporated into a new production cycle of RPUF-C-type materials in the form, once again, of fillers. The results can be seen in Table S7; the amount of catalyst was adjusted in the final formula.

During the preparation of the polyol, no change in fluidity was observed; the polyol remained liquid after the addition of the paste; however, the recycled paste had an accelerating effect on the RPUF-C reaction, as the reaction times were shortened significantly. The reactivity increased as the paste concentration increased (standard time for PUF-C is 60 s). In the reaction initiation times, it was observed that an increase in the amount of paste led to an advance in the reaction initiation time, indicating, once again, that this substance could have some catalytic effect on the system. It was also observed that it does not seem to activate the reaction on its own, as in the experiments without catalyst, no foaming or polymerization of the material was observed until two days after mixing. In the other cases, the expansion during the polymerization of the PUR was disorderly, with different reaction starting points in the mixture and uneven curing, which resulted in an inhomogeneous and unstructured material (see Figure S18, left), which made it impossible to measure the curing time of the PUR accurately.

However, given that the reaction onset times were not very fast, tests were carried out on plates with the Ref. [3] formulation, which was the least reactive, but once the manual agitation was completed, there was an increase in the viscosity of the mixtures that made manual injection difficult; therefore, the mold could not be filled completely, resulting in incomplete plates of very low density (see Figure S18 right).

## 4. Conclusions

Two pre-consumer PU wastes were selected, one aromatic and the other aliphatic. These materials were conditioned by size reduction. PU wastes were characterized by TG, DSC, X-ray fluorescence, ATR-IR, and calcination. The IR spectra of the ashes resulting from the calcination of the residues showed silica-silicate bands. X-ray fluorescence showed a higher amount of Ti in the case of RPUF-C and the presence of Sn and Ba. ATR-IR showed typical PU peaks of aliphatic (RPUF-C) and aromatic peaks (RPUF-B).

Reactions without catalyst were studied, showing that RPUF-B presented high conversions (>90%) at 2 h. The need to introduce a catalyst to RPUF-C was observed. NaOH catalyzed the reaction slightly, improving the conversion of PU, as did Na acetate. The Na acetate catalyst made the reaction unstable during the first minutes and, in the case of RPUF-C, significantly reduced the temperature. For this residue, the use of conventional catalysts such as DEA was studied, and their use significantly reduced the conversion.

The use of EG versus DEG allowed, in general, obtaining more manageable glycolyzed products, with lower viscosity, which made possible the subsequent filtering and/or centrifuging stages. In the case of RPUF-C, DEG is a solvent that prevents the glycolysis reaction, unlike EG. As for temperature, the greatest effect was observed for the RPUF-C residue. The depolymerization reactions were optimized in terms of times and granulometry.

The analysis of the GPCs performed at the different times and granulometries indicated that in the case of the RPUF-B residue, the reaction is practically instantaneous. This indicates that as the PU residue is being fed to the solvent, which is in the reactor at boiling conditions, and with the appropriate catalyst, depolymerization of the PU occurs immediately. Similarly, the results of the GPCs showed that the generation of polyols is very similar at all times at relevant concentrations. In both cases (RPUF-B and RPUF-C), 5 mm grinding was the optimum one as there was no improvement when the size was lower than 5 mm. In the case of RPUF-C, the best results were obtained with the 5 mm grinding at 90 min of glycolysis, and with the 2 mm grinding, the best results were obtained at 90 min of glycolysis; with respect to the 500-micron grinding, the best results were obtained at 120 min of glycolysis. Therefore, from this study, it can be concluded that the optimal glycolysis reaction for the RPUF-C residue is with a 5 mm grind for 90 min.

Furthermore, the GPCs performed indicated that polyols of a similar structure to the original ones used in the formulations of the initial polyurethanes (Lupranols) were obtained, since equivalent peaks were obtained at similar retention times comparing the GPCs of the reaction products with those of the commercial reference polyols. Noteworthy also is the presence of other peaks in the GPCs, which indicated the presence of reaction products, which were other compounds in addition to the starting polyols. The chemical structure of these compounds is associated with the isocyanate used in the manufacture of polyurethane, in addition to fillers and additives commonly used in such manufacture.

Regarding the introduction of the GP obtained in the new RPUF formulations, in view of the results in the aromatic system (RPUF-B), it seems more convenient to introduce the recycled materials in aliphatic PUR systems (RPUF-C), whose reactivity is lower. The amount of catalyst added during the depolymerization reaction was rather small; the possible influence of the residue catalyst was uncertain and, in any case, was overlapped by the influence of amines and fillers.

There was 15% of GP-B introduced into the RPUF-C formulation, resulting in an inhibition of the reaction times; GP-C only had 5% due to the high reactivity observed, probably due to the high amount of aluminum that comes from the very high percentage of aluminum hydroxide included in the initial formulation, since this is used to provide the polyurethane with flame-retardant properties. However, it is not known to what extent this aluminum can catalyze the synthesis of PUR since, normally, the catalysts used are usually organometallic complexes of Zn, Co, Bi, Sn, etc., with empty electronic orbitals. Another interesting possible application of these polyols is their use as catalysts to replace the currently used DBTL, for which alternatives are being sought due to its toxicity. It is difficult to find a compound that initiates the reaction as fast as DBTL, so this green polyol could be used for this purpose.

Besides, the introduction of the recycled paste from the glycolysis process was also studied, resulting also in the speed up of the RPUF-C polymerization system. More trials need to be performed to achieve a good plate.

## Figures and Tables

**Table 1:** Current strategies for recycling RPUF.

Treatment	Technology	Main Waste Source	Obtained Products	Added Value of Products	Applied Level	Cost	Level of Industrialization	Research Needs
Mechanical recycling	Milling, mix with a binder	Pre-consumer	Pellets, flakes, inert filler, parts	Low	Medium	Low	High	Low
Chemical recycling	Hydrolysis	Pre-, post-consumer	Polyols, amine intermediates	High	Low	Medium	Low	High
	Aminolysis	Pre-consumer foams	Amines, alcohols	High	Low	Medium	Low	High
	Glycolysis	Pre-, post-consumer foams	Polyols	High	Low	Medium	Low	High
	Alcoholysis	Pre-, post-consumer	Polyols, urethane products	High	Low	Medium	Low	High
	Gasification	Pre, post-consumer	Synthesis gas, ash	Medium-high	Medium	High	High	Medium
	Pyrolysis	Pre-, post-consumer	Oil, gas, char	Medium-high	Low	High	Medium	Medium
Energy recovery	Combustion	Post-consumer	Energy	Low	Medium	High	High	Low
Biological degradation	Micro-organisms, enzymes or fungi	Pre-, post-consumer	Polyols, amines, carboxylic acids	High	Low	Medium	Low	High
Landfilling	Landfilling	Pre-, post-consumer	None	None	High	Low	High	None

**Table 2:** Conditions of the glycolysis reactions, RPUF-B.

Glycolysis Reaction Code	Particle Size (mm)	Solvent	Solvent:PU (g:g)	Temperature (°C)	Catalyst	Catalyst:PU (mol:g)	Time (h)	Conversion (%) g Reacted/g PUF
R5	2	EG	4:1	198	NaOH	0.002	2	98
R6	2	EG	4:1	180	NaOH	0.002	2	96
R7	2	DEG	4:1	198	NaOH	0.002	2	98
R12	2	EG	4:1	198	NaOH	0.001	2	98
R19	2	EG	4:1	198	-	-	2	93
R20	2	EG	4:1	198	Na Acetate	0.002	2	98
R39	2	EG	4:1	198	NaOH	0.002	2	97
R44	2	EG	4:1	198	NaOH	0.002	2	98
R46	2	EG	4:1	198	NaOH	0.002	2	98
R52	2	EG	4:1	198	NaOH	0.002	2	98
R53	0.5	EG	4:1	198	NaOH	0.002	2	98
R54	5	EG	4:1	198	NaOH	0.002	2	98
R55	2	EG	4:1	198	NaOH	0.002	2	98
R56	0.5	EG	4:1	198	NaOH	0.002	2	98
R57	5	EG	4:1	198	NaOH	0.002	2	98
R58	2	EG	4:1	198	NaOH	0.002	2	98

**Table 3:** Concentration in percentage of each of the components of the product of the reaction 56 at different times: recovered polyol, by-products, glycolysis agent.

Concentration (%wt)	1 min	3 min	5 min	7 min	10 min	15 min	30 min	45 min	60 min	90 min	120 min
Recovered Polyols	35.8	36.4	36.5	36.4	34	36.3	36.8	37.3	36.5	37.9	38.1
Subproducts	15.1	15.3	16	15.9	14.9	13.9	14.5	15	14.3	15.3	15.6
Glycolysis agent	49.1	48.3	47.5	47.7	51.1	49.8	48.7	47.7	49.2	46.8	46.3

**Table 4:** Concentration in percentage of each of the components of the product of the reactions R53, R54, R55, R56, R57, and R58 at different times: recovered polyol, by-products, glycolysis agent.

Recovered Polyol (%wt)	1 min	3 min	5 min	7 min	10 min	15 min	30 min	45 min	60 min	90 min	120 min
R53, R56 500 µm	35.8	36.4	36.5	36.4	34	36.3	36.8	37.3	36.5	37.9	38.1
R55, R58 2 mm	37.1	36.2	35.9	36.3	36.1	38.7	38.5	38.5	38.1	39.2	38.3
R54, R57 5 mm	36.1	35.8	35.5	35.2	35.1	35.3	36.2	35.2	36.5	35.6	36.1

**Table 5:** Properties of the Green Polyols (GPs) obtained and properties of the Commercial Polyol (CP).

Polyol Code	IOH (mg KOH/g)	IA (mg KOH/g)	µ (cP)
GP-B	692 ± 85	8.7 ± 0.09	17,820 ± 970
GP-C	643 ± 95	4.48 ± 0.42	10,720 ± 610
CP	395 ± 65	0.09 ± 0.02	1690 ± 35

**Table 6:** Results of the reaction onset and curing time of GPs in the RPUF-C formulations.

Type of GP	Amount of GP in the RPUF-C	Reaction Onset Time *	Curing Time **
--	--	50–60 s	1 min 30 s
GP-B	5%	64 s	1 min 53 s
GP-B	10%	88 s	2 min 24 s
GP-C-2 mm	5%	50 s	1 min 15 s
GP-C-5 mm	5%	55 s	1 min 45 s

* Reaction onset time = time taken for the foam to start once all reagents are added. ** Curing time = time for curing once foaming has started.

## Supplementary Materials

The following Supporting Information can be downloaded at: https://www.mdpi.com/article/10.3390/polym14142936/s1, Figure S1: Spectra obtained by ATR-FTIR spectroscopy of RPUF-B (left) and RPUF-C (right) residues; Figure S2: Spectra obtained by ATR-FTIR spectroscopy of the ashes resulting from the calcination of RPUF residues. RPUF-B (left) and RPUF-C (right); Figure S3: ATR-IR spectrum of comercial reference polyols; Figure S4: ATR-IR spectrum of Reaction 5 (green) compared wit EG (black) and PURF-B (blue); Figure S5: ATR-IR spectrums of the products of Reactions 20 and 19 compared with and RPUF-B (red); Figure S6: IR spectra of the refined products of the reactions R5 and R8 at 200 ºC, 2 h, PUR/solvent ratio = 1/4, and NaOH (0.002 mol/g): (a) R5, PURF-B and (b) R8, PURF-C; Figure S7: IR spectra of the reactions at 200 ºC, 2 h and PUR/solvent ratio = 1/4, after removal of the EG by distillation: RPUF-B (a) with Na acetate (0.002 mol/g), (b) without catalyst, (c) with NaOH (0.002 mol/g); RPUF-C (d) with Na acetate (0.002 mol/g) and (e) with NaOH (0.002 mol/g); Figure S8: GPC of Lupranol 3300, one of the polyols used in PURF-B parts and its components concentration; Figure S9: GPC of Lupranol 3402, one of the polyols used in PURF-B parts and its components concentration; Figure S10: GPC of Lupranol 3422, one of the polyols used in PURF-B parts and its components concentration; Figure S11: GPC of Lupranol 3505-1, polyol used in PURF-C parts and its components concentration; Figure S12: GPC of reaction 56, min 1, 3. 5, 7 and 10; Figure S13: Screening (GPC) of polyols with the residue RPUF-C at 500 microns; Figure S14: Screening (GPC) of polyols with the residue RPUF-C at 2 mm; Figure S15: Screening (GPC) of polyols with the residue RPUF-C at 5 mm; Figure S16: Images of GP-B (left) and GP-C obtained with the initial waste at 500 microns, 5 mm and 2 mm (right); Figure S17: Some defects found in plates of GP-B at 10% (left) and GP-C at 5% (right); Figure S18: Free foaming for ref. [3] (up left), ref. [4] (up right), ref. [5] (down left) and ref. [6] (down right). Plate for ref. [3] (right); Table S1. Conditions of the glycolysis reactions RPUF-C; Table S2. Inorganic matter content after muffle calcination of the wastes; Table S3. TG and dTG curves of the residues RPUF-B and RPUF-C; Table S4. Percentage by weight of elements present in RPUF residues analyzed by XRF; Table S5. Results of the quantity of polyols obtained in each of the glycolysis reactions for 500 microns, 2 mm and 5 mm RPUF-C waste; Table S6. Hydroxyl number of the polyols obtained; Table S7. Formulations with recycled paste.
