# Supplementary material for: Recovery of Green Polyols from Rigid Polyurethane Waste by Catalytic Depolymerization
Source: Polymers (Basel). 2022 Jul 20;14(14):2936. doi: 10.3390/polym14142936 (PMC9319047; doi:10.3390/polym14142936)
Supplement: Supplementary file 1 [file polymers-14-02936-s001.zip › polymers-1789036-supplementary.pdf]

# Supporting information

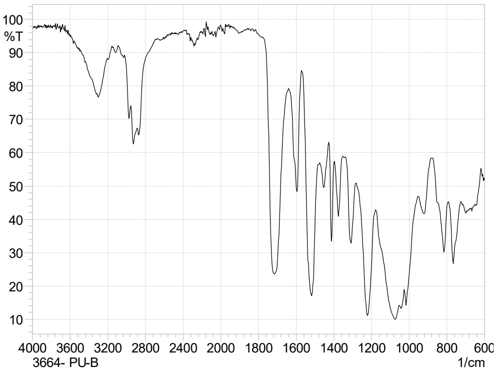


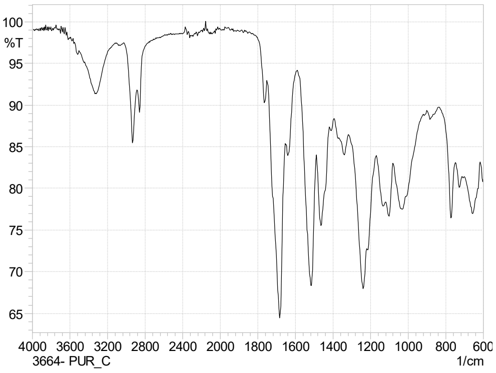


Figure S1. Spectra obtained by ATR-FTIR spectroscopy of RPUF-B (left) and RPUF-C (right) residues.

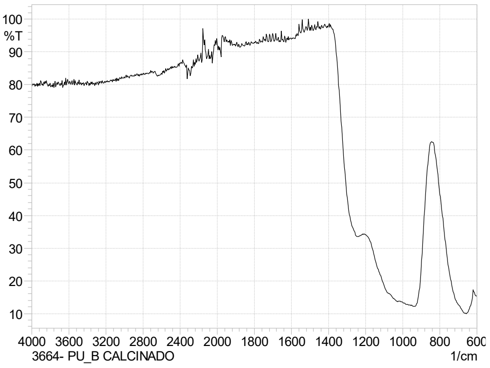


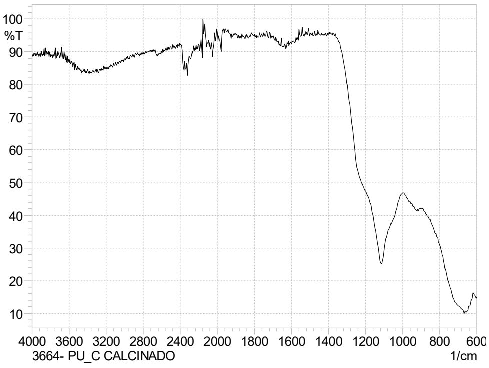


Figure S2.Spectra obtained by ATR-FTIR spectroscopy of the ashes resulting from the calcination of RPUF residues. RPUF-B (left) and RPUF-C (right).

| | | |
|---|---|---|
| 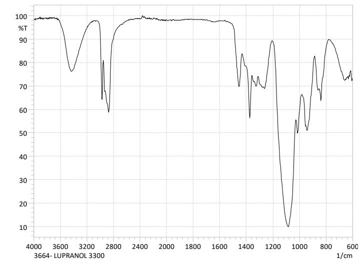 | 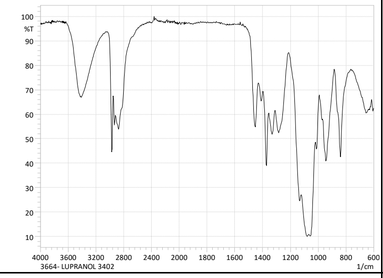 | 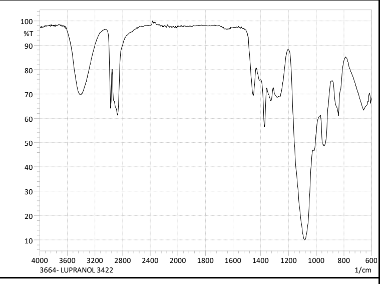 |
| Lupranol 3300 | Lupranol 3402 | Lupranol 3422 |

Figure S3. ATR-IR spectrum of comercial reference polyols.

| | | |
|---|---|---|
| 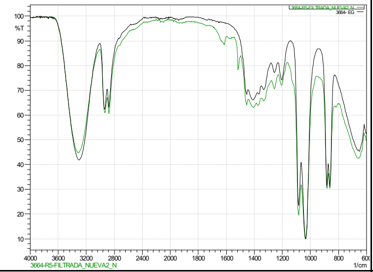 | 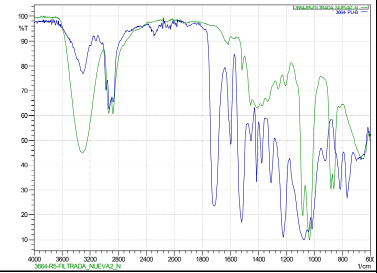 | 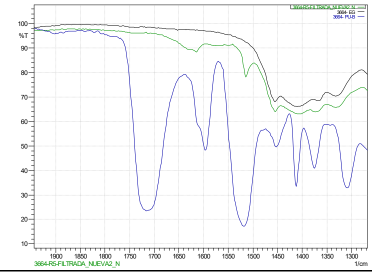 |
| R5 – 120 and EG | R5 – 120 and PURF-B | R5 – 120, EG and PURF-B (Zoom) |

Figure S4. ATR-IR spectrum of Reaction 5 (green) compared wit EG (black) and PURF-B (blue).

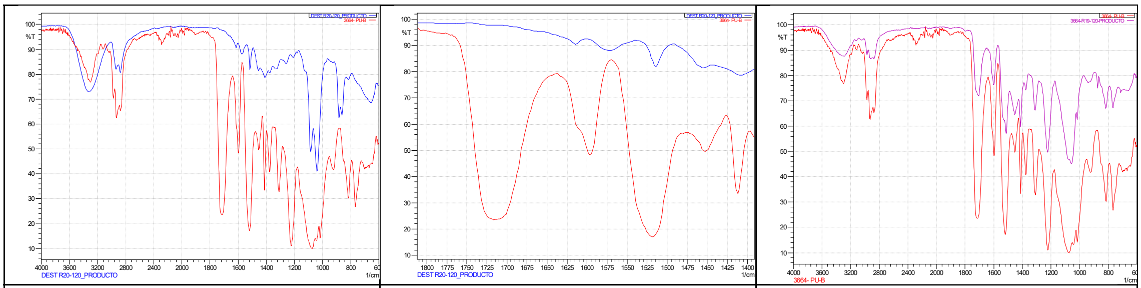

| R20-120 distilled and RPUF-B | R20-120 distilled and RPUF-B (zoom) | R19-120 and RPUF-B |
|---|---|---|

Figure S5. ATR-IR spectrums of the products of Reactions 20 and 19 compared with and RPUF-B (red).

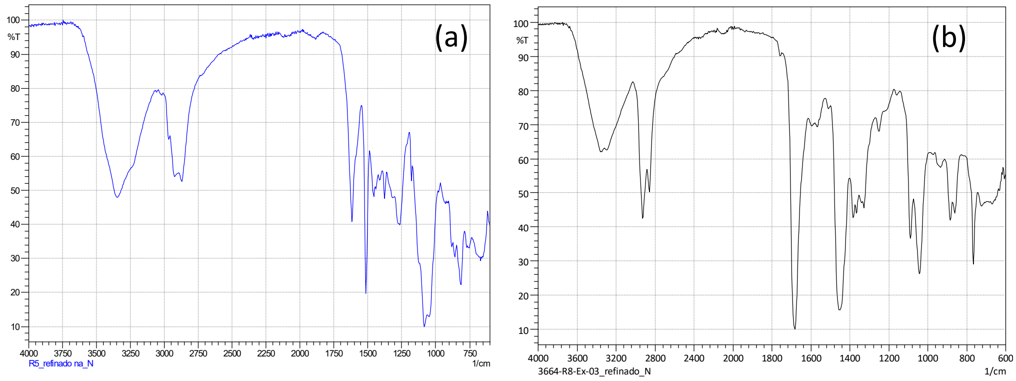


Figure S6. IR spectra of the refined products of the reactions R5 and R8 at 200ºC, 2h, PUR/solvent ratio = 1/4, and NaOH (0.002 mol/g): (a) R5, PURF-B and (b) R8, PURF-C.

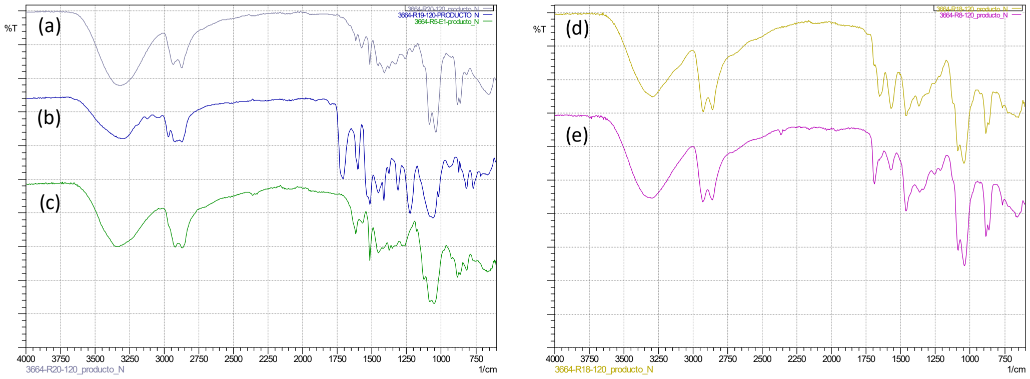


Figure S7. IR spectra of the reactions at 200ºC, 2h and PUR/solvent ratio = 1/4, after removal of the EG by distillation: RPUF-B (a) with Na acetate (0.002 mol/g), (b) without catalyst, (c) with NaOH (0.002 mol/g); RPUF-C (d) with Na acetate (0.002 mol/g) and (e) with NaOH (0.002 mol/g).

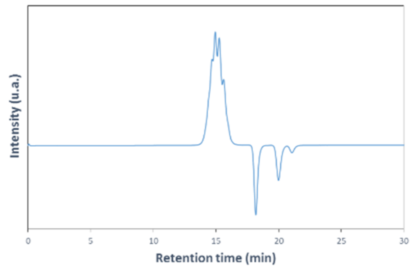


| CONCENTRATION |
|---|
| 27.321 |
| 26.572 |
| 25.934 |
| 20.172 |

Figure S8. GPC of Lupranol 3300, one of the polyols used in PURF-B parts and its components concentration.

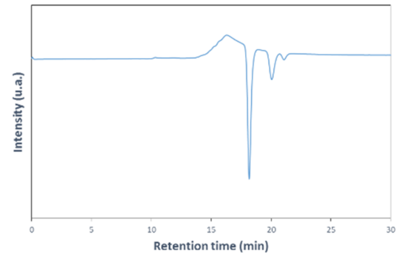


| CONCENTRATION |
|---|
| 69.416 |
| 30.584 |

Figure S9. GPC of Lupranol 3402, one of the polyols used in PURF-B parts and its components concentration

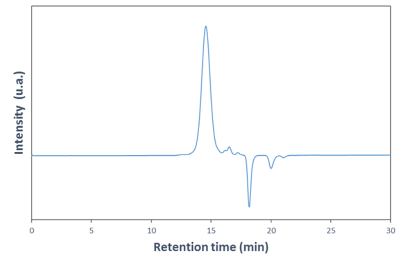


| CONCENTRATION |
|---|
| 89.504 |
| 1.227 |
| 2.553 |
| 6.717 |

Figure S10. GPC of Lupranol 3422, one of the polyols used in PURF-B parts and its components concentration

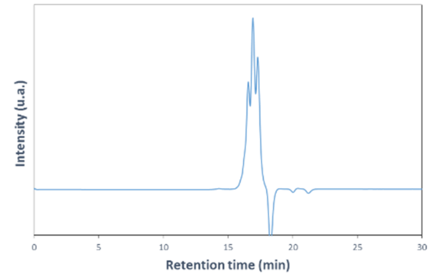


| CONCENTRATION |
|---|
| 27.687 |
| 36.170 |
| 36.141 |

Figure S11. GPC of Lupranol 3505-1, polyol used in PURF-C parts and its components concentration

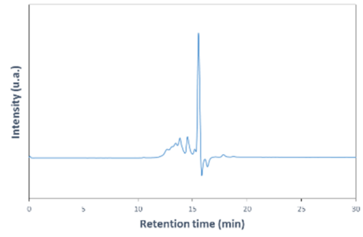


R-56-1

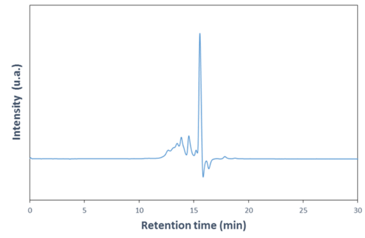


R-56-3

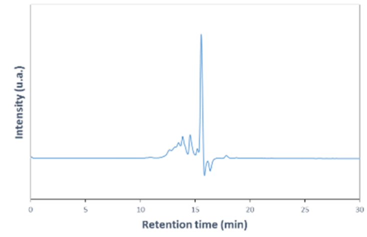


R-56-5

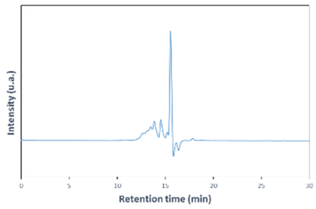


R-56-7

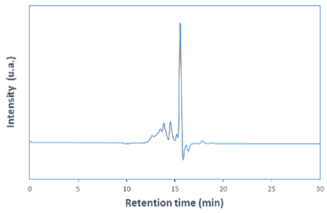


R-56-10

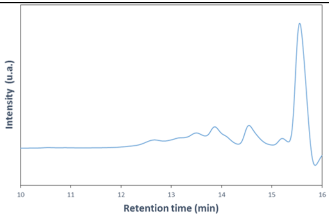


R-56R-10 Detail

Figure S12. GPC of reaction 56, min 1, 3. 5, 7 and 10.

| Chemical product | mg/Kg sample | % | mg/Kg sample | % | mg/Kg sample | % | mg/Kg sample | % |
|---|---|---|---|---|---|---|---|---|
| Furan, tetrahydro-2-methyl- | 3996,172648 | 0,399617265 | 4048,250023 | 0,404825002 | 4009,571885 | 0,400957189 | 4180,405934 | 0,418040593 |
| Peroxide, dipropyl | 79391,58525 | 7,939158525 | 58581,83635 | 5,858183635 | 59568,40671 | 5,956840671 | 87345,41346 | 8,734541346 |
| 2-Furanol, tetrahydro- | 140616,5221 | 14,06165221 | 128928,7775 | 12,89287775 | 120093,2261 | 12,00932261 | 138877,9914 | 13,88779914 |
| 1,3-Propanediol | 1894,437303 | 0,18944373 | 1090,622085 | 0,109062209 | 1642,697729 | 0,164269773 | 2428,196503 | 0,24281965 |
| Butanoic acid, 4-hydroxy- | 47219,85388 | 4,721985388 | 26544,07706 | 2,654407706 | 23861,83002 | 2,386183002 | 27402,23655 | 2,740223655 |
| Furan, tetrahydro-2-methyl- | 0 | 0 | 23303,82875 | 2,330382875 | 28932,56526 | 2,893256526 | 38156,34828 | 3,815634828 |
| 1,3-Propanediol, 2-ethyl-2-(hydroxymethyl)- | 1183,798888 | 0,118379889 | 4420,287197 | 0,44202872 | 3464,91991 | 0,346491991 | 3690,213684 | 0,369021368 |
| **3-Amino-4,5,6,7,8,9-hexahydrocyclooct(c)isoxazole** | | | | | | | 763,5831038 | 0,07635831 |
| **-Pyrrolizine-7-methanol, 2,3,5,7a-tetrahydro-1-hydroxy-, (1S-cis)-** | | | | | | | 30,0198052 | 0,003001981 |
| 1,3-Propanediol, 2-ethyl-2-(hydroxymethyl)- | 514,4212085 | 0,051442121 | 5089,753363 | 0,508975336 | 10126,51682 | 1,012651682 | 2341,890758 | 0,234189076 |
| Cyclopropanetetradecanoic acid, 2-octyl-, methyl ester | | | 0 | 0 | 979,8361515 | 0,097983615 | 11966,40967 | 1,196640967 |
| Octaethylene glycol monododecyl ether | | | 0 | 0 | 968,7880511 | 0,096878805 | 1239,990102 | 0,12399901 |
| **1H-Pyrrole, 1-butyl-** | | | 1054,248391 | 0,105424839 | 3988,857385 | 0,398885738 | 1190,305435 | 0,119030544 |
| Hexanedioic acid, bis(2-ethylhexyl) ester | | | 0 | 0 | 0 | 0 | 7127,106069 | 0,712710607 |
| | 274816,7913 | 27,48167913 | 253061,6807 | 25,30616807 | 257637,216 | 25,7637216 | 326740,1107 | 32,67401107 |

Figure S13. Screening (GPC) of polyols with the residue RPUF-C at 500 microns

| | 30 min | | 60 min | | 90 min | | 120 min | |
|---|---|---|---|---|---|---|---|---|
| **Chemical product** | mg/Kg sample | % | mg/Kg sample | % | mg/Kg sample | % | mg/Kg sample | % |
| Furan, tetrahydro-2-methyl- | 6947,569339 | 0,694756934 | 7050,718717 | 0,705071872 | 7106,461941 | 0,710646194 | 4587,935227 | 0,458793523 |
| Peroxide, dipropyl | 64867,64566 | 6,486764566 | 73528,27035 | 7,352827035 | 68579,17733 | 6,857917733 | 65360,54688 | 6,536054688 |
| 2-Furanol, tetrahydro- | 106551,0563 | 10,65510563 | 124006,6168 | 12,40066168 | 116844,5741 | 11,68445741 | 87332,14442 | 8,733214442 |
| 2-Furanol, tetrahydro- | | | | | | | 60950,04746 | 6,095004746 |
| 2-Furanol, tetrahydro- | 245063,6248 | 24,50636248 | 198977,019 | 19,8977019 | 198846,933 | 19,8846933 | 27079,0621 | 2,70790621 |
| 1,3-Propanediol | 8047,359881 | 0,804735988 | 7482,117292 | 0,748211729 | 8112,027929 | 0,811202793 | 2777,037268 | 0,277703727 |
| Butyrolactone | 122089,687 | 12,2089687 | 105135,5173 | 10,51355173 | 105881,8447 | 10,58818447 | 0 | 0 |
| Furan, tetrahydro-2-methyl- | 56790,32907 | 5,679032907 | 85024,78021 | 8,502478021 | 124277,2049 | 12,42772049 | 99586,89143 | 9,958689143 |
| 1,3-Propanediol, 2-ethyl-2-(hydroxymethyl)- | 1567,762112 | 0,156776211 | 1909,118294 | 0,190911829 | 4221,427035 | 0,422142703 | 3227,514786 | 0,322751479 |
| 1,3-Propanediol, 2-ethyl-2-(hydroxymethyl)- | | | 3629,914692 | 0,362991469 | 7095,149585 | 0,709514959 | 10699,19631 | 1,069919631 |
| Cyclopropanetetradecanoic acid, 2-octyl-, methyl ester | | | | | | | 357,5480273 | 0,035754803 |
| Octaethylene glycol monododecyl ether | | | | | | | 710,1276505 | 0,071012765 |
| **1H-Pyrrole, 1-butyl-** | | | | | | | 2374,220203 | 0,23742202 |
| | 611925,0341 | 61,19250341 | 606744,0727 | 60,67440727 | 640964,8004 | 64,09648004 | 350901,1796 | 35,09011796 |

Figure S14. Screening (GPC) of polyols with the residue RPUF-C at 2 mm

| | 30 min | | 60 min | | 90 min | | 120 min | |
|---|---|---|---|---|---|---|---|---|
| **Chemical product** | mg/Kg sample | % | mg/Kg sample | % | mg/Kg sample | % | mg/Kg sample | % |
| Furan, tetrahydro-2-methyl- | 3175,150577 | 0,317515058 | 2844,901109 | 0,284490111 | 3373,524697 | 0,33735247 | 2360,116965 | 0,236011696 |
| Peroxide, dipropyl | 59250,70032 | 5,925070032 | 101718,0511 | 10,17180511 | 55579,87916 | 5,557987916 | 66912,35186 | 6,691235186 |
| Toluene | 9460,265751 | 0,946026575 | 0 | 0 | 0 | 0 | 0 | 0 |
| 2-Furanol, tetrahydro- | 104575,4285 | 10,45754285 | 114942,5579 | 11,49425579 | 98274,33565 | 9,827433565 | 98095,76493 | 9,809576493 |
| 1,3-Propanediol | 104,8407983 | 0,01048408 | 0 | 0 | 0 | 0 | 0 | 0 |
| Butanoic acid, 4-hydroxy- | 11389,13446 | 1,138913446 | 10994,6978 | 1,09946978 | 9621,739728 | 0,962173973 | 10373,26248 | 1,037326248 |
| Furan, tetrahydro-2-methyl- | 21829,67221 | 2,182967221 | 25142,75111 | 2,514275111 | 21294,75696 | 2,129475696 | 23303,00791 | 2,330300791 |
| 1,3-Propanediol, 2-ethyl-2-(hydroxymethyl)- | 7983,554621 | 0,798355462 | 6929,695152 | 0,692969515 | 8800,993015 | 0,880099301 | 4235,142966 | 0,423514297 |
| 1,3-Propanediol, 2-ethyl-2-(hydroxymethyl)- | 9645,658904 | 0,96456589 | 6641,483922 | 0,664148392 | 14674,9614 | 1,46749614 | 18760,63495 | 1,876063495 |
| Cyclopropanetetradecanoic acid, 2-octyl-, methyl ester | | 0 | | 0 | 1679,301084 | 0,167930108 | 2332,652654 | 0,233265265 |
| Octaethylene glycol monododecyl ether | 821,8464656 | 0,082184647 | 290,3104066 | 0,029031041 | 1815,623209 | 0,181562321 | 3159,153696 | 0,31591537 |
| **1H-Pyrrole, 1-butyl-** | | 0 | 0 | 0 | 0 | 0 | 0 | 0 |
| Hexanedioic acid, bis(2-ethylhexyl) ester | 0 | 0 | 0 | 0 | | | 2682,643393 | 0,268264339 |
| Phenol, 2,2'-methylenebis[6-(1,1-dimethylethyl)-4-methyl- | 0 | 0 | 601,5494312 | 0,060154943 | 0 | 0 | 0 | 0 |
| Bis(2-ethylhexyl) phthalate | 0 | 0 | 6517,296736 | 0,651729674 | 12430,07922 | 1,243007922 | 73235,55049 | 7,323555049 |
| | 228236,2526 | 22,82362526 | 276623,2947 | 27,66232947 | 227545,1941 | 22,75451941 | 305450,2823 | 30,54502823 |

Figure S15. Screening (GPC) of polyols with the residue RPUF-C at 5 mm

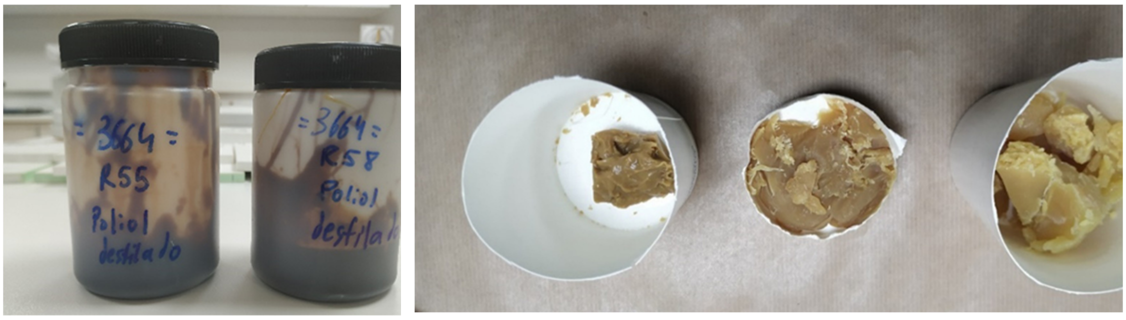


Figure S16. Images of GP-B (left) and GP-C obtained with the initial waste at 500 microns, 5 mm and 2 mm (right)

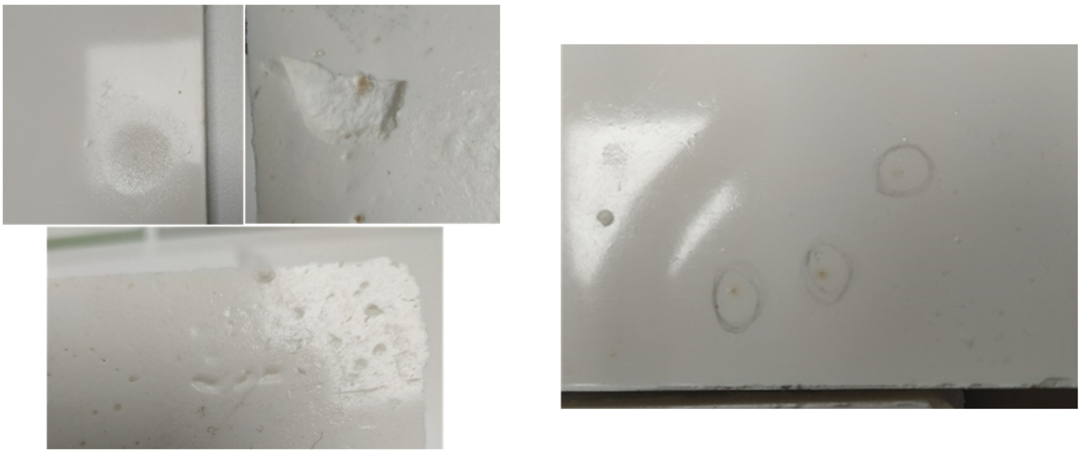

Figure S17. Some defects found in plates of GP-B at 10% (left) and GP-C at 5% (right)

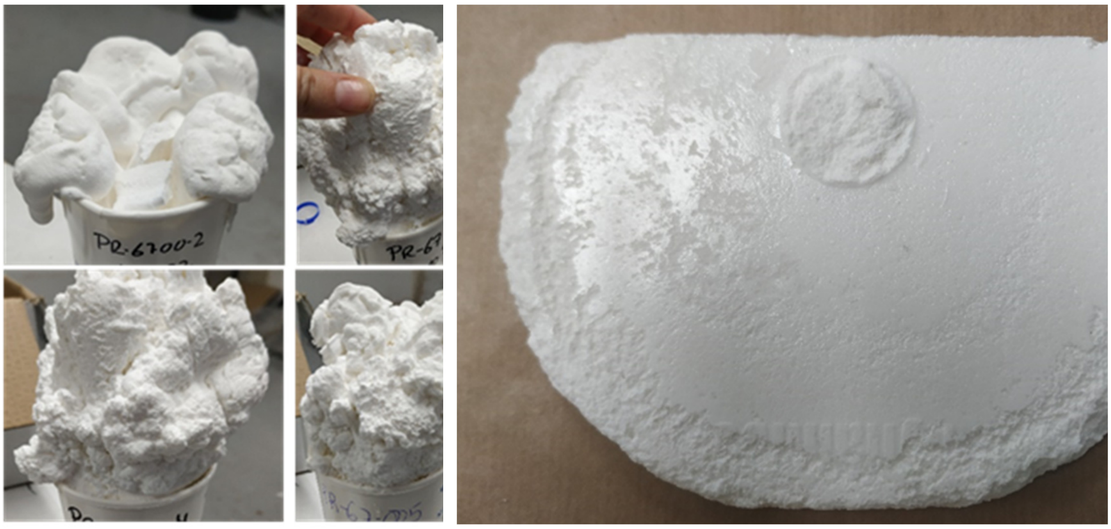

Figure S18. Free foaming for ref. 3 (up left), ref. 4 (up right), ref. 5 (down left) and ref. 6 (down right). Plate for ref. 3 (right)

**Table S1**. Conditions of the glycolysis reactions RPUF-C.

| Glycolysis Reaction Code | Particle size [mm]. | Solvent | Solvent:PU [g:g]. | Temperature [°C] | Catalyst | Catalyst :PU [mol:g] | Time [h] | Conversion (%) g reacted /g PURF |
|---|---|---|---|---|---|---|---|---|
| R8 | 2 | E.G. | 4:1 | 198 | NaOH | 0.002 | 2 | 48 |
| R9 | 2 | D.E.G. | 4:1 | 200 | NaOH | 0.002 | 2 | 0 |
| R13 | 2 | E.G. | 4:1 | 198 | NaOH | 0.001 | 2 | 64 |
| R15 | 2 | E.G. | 4:1 | 198 | - | 0.002 | 2 | 47 |
| R18 | 2 | E.G. | 4:1 | 198 | Na Acetate | - | 2 | 69 |
| R24 | 2 | E.G. | 4:1 | 180 | NaOH | 0.002 | 2 | 0 |
| R25 | 2 | E.G. | 2:1 | 198 | NaOH | 0.002 | 2 | 0 |
| R27 | 2 | E.G. | 4:1 | 180 | Na Acetate | 0.002 | 2 | 0 |
| R34 | 2 | E.G. | 4:1 | 198 | DEA | 0.002 | 2 | 38 |
| R36 | 2 | E.G. | 4:1 | 198 | NaOH | 0.002 | 2 | 63 |
| R40 | 2 | E.G. | 4:1 | 198 | NaOH | 0.002 | 2 | 53 |
| R41 | 5 | E.G. | 4:1 | 198 | Na Acetate | 0.002 | 5 | 77 |
| R45 | 2 | E.G. | 4:1 | 198 | NaOH | 0.002 | 2 | 100 |
| R48 | 2 | E.G. | 4:1 | 198 | NaOH | 0.002 | 2 | 94 |

| | | | | | | | | |
|---|---|---|---|---|---|---|---|---|
| R49 | 5 | E.G. | 4:1 | 198 | NaOH | 0.002 | 2 | 90 |

**Table S2**. Inorganic matter content after muffle calcination of the wastes.

| Waste type | RPUF-B | RPUF-C |
|---|---|---|
| Inorganic fillers (%) | 1.2 | 9.3 |
| Standard deviation | 0.0 | 0.0 |

**Table S3.** TG and dTG curves of the residues RPUF-B and RPUF-C.

| Waste Type | Degradation range [°C] | Solid residue [°C] | Maximum degradation T [°C] | Glass transition T [°C] |
|---|---|---|---|---|
| RPUF-B | 250-540 | 14.8 | 349 | 78 |
| RPUF-C | 250-490 | 11.4 | 393 | 66 |

**Table S4.** Percentage by weight of elements present in RPUF residues analyzed by XRF.

| Element/ % Weight | RPUF-B | RPUF-C |
|---|---|---|
| Ag, As, Au, Bi, Br, Ca, Cd, Cl, Co, Cr, Cu, Fe, Hg, Mn, Mo, Ni, Pb, Sb, Se, Sr, Zn, Zr | 0.0 | 0.0 |
| Ba | 0.0 | 0.1 |
| Sn | 0.0 | 0.1 |
| Ti | 0.4 | 1.3 |
| Total | 0.4 | 1.5 |
| Standard Deviation (%) | 0.0 | 0.3 |

**Table S5.** Results of the quantity of polyols obtained in each of the glycolysis reactions for 500 microns, 2 mm and 5 mm RPUF-C waste

| | Glycolysis reaction time | | |
|---|---|---|---|
| RPUF-C wasted | 60 min | 90 min | 120 min |
| 500 microns | 60,51 g | 67,51 g | 75,53 g |
| 2 mm | 43,52 g | 62,73 g | 42,36 g |
| 5 mm | 25,34 g | 68,14 g | 65,35 g |

**Table S6.** Hydroxyl number of the polyols obtained

| Polyols | Hydroxyl number (mgKOH/g) |
|---|---|
| 500 microns/90 min | 632 |
| 500 microns/120 min | 682 |
| 2mm/90min | 815 |
| 2mm/120 min | 929 |

| Polyols | Hydroxyl number (mgKOH/g) |
|---|---|
| 5 mm/ 5 min | 764 |
| 5 mm/ 30 min | Insoluble, no measure |
| 5 mm/ 60 min | |
| 5 mm/ 90 min | 673 |
| 5 mm/ 120 min | 821 |

**Table S7.** Formulations with recycled paste.

| Reference sample | Recycled paste in the total formulation | Polyol for RPUF-C | Catalyst | Espumation time (s) |
|---|---|---|---|---|
| 1 | 0% | 100% | 0,16% | 60s |
| 2 | 5,0% | 95,0% | 0,00% | No activación |
| 3 | 5,0% | 95,0% | 0,16% | 100s |
| 4 | 5,0% | 95,0% | 0,32% | 41s |
| 5 | 10,0% | 90,0% | 0,16% | 45s |

| | | | | |
|---|---|---|---|---|
| 6 | 15,0% | 85,0% | 0,16% | 42s |
| 7 | 30,0% | 70,0% | 0,16% | 0s |
